# Development of a multi-species SNP array for serrasalmid fish *Colossoma macropomum* and *Piaractus mesopotamicus*

**DOI:** 10.1038/s41598-021-98885-x

**Published:** 2021-09-29

**Authors:** Vito A. Mastrochirico-Filho, Raquel B. Ariede, Milena V. Freitas, Carolina H. S. Borges, Lieschen V. G. Lira, Natália J. Mendes, John F. G. Agudelo, Pablo Cáceres, Milthon H. M. Berrocal, Gustavo A. L. Sucerquia, Fabio Porto-Foresti, José M. Yáñez, Diogo T. Hashimoto

**Affiliations:** 1 São Paulo State University (Unesp), Aquaculture Center of Unesp, Jaboticabal, SP 14884-900 Brazil; 2 Facultad de Ciencias Veterinarias y Pecuarias, Universidad de Chile, Santiago, Chile; 3 Facultad de Zootecnia, Universidad Nacional Agraria de la Selva, Tingo Maria, Peru; 4 Facultad de Ciencias Agrarias, Universidad de Antioquia, Medellín, Colombia

**Keywords:** Computational biology and bioinformatics, Genetics, Molecular biology

## Abstract

Scarce genomic resources have limited the development of breeding programs for serrasalmid fish *Colossoma macropomum* (tambaqui) and *Piaractus mesopotamicus* (pacu), the key native freshwater fish species produced in South America. The main objectives of this study were to design a dense SNP array for this fish group and to validate its performance on farmed populations from several locations in South America. Using multiple approaches based on different populations of tambaqui and pacu, a final list of 29,575 and 29,612 putative SNPs was selected, respectively, to print an Axiom AFFYMETRIX (THERMOFISHER) SerraSNP array. After validation, 74.17% (n = 21,963) and 71.25% (n = 21,072) of SNPs were classified as polymorphic variants in pacu and tambaqui, respectively. Most of the SNPs segregated within each population ranging from 14,199 to 19,856 in pacu; and from 15,075 to 20,380 in tambaqui. Our results indicate high levels of genetic diversity and clustered samples according to their hatchery origin. The developed SerraSNP array represents a valuable genomic tool approaching in-depth genetic studies for these species.

## Introduction

Tambaquis, pacus and piranhas are included in the family Serrasalmidae (Ostariophysi: Characiformes)^1^, and they are broadly distributed throughout the major river systems of South America^2^. Serrasalmids (round-shaped fish) are a diverse group comprising 101 valid species, represented by a variety of feeding strategies and associated morphological adaptations^3^. While piranhas are popularly known for voracious and carnivorous behavior, tambaquis and pacus are omnivorous, possessing special teeth to eat fruit and seeds, and have a great ability to harness natural food sources (zooplankton). Beyond their feeding habitats, these species have excellent traits of economic interest for aquaculture including high growth performance, ease of reproduction and market appreciation^4–6^.

Tambaqui (*Colossoma macropomum*), pacu (*Piaractus mesopotamicus*) and pirapitinga (*Piaractus brachypomus*) are the major serrasalmid fish produced by the aquaculture industry in South America^4^. Tambaqui and pirapitinga are mainly farmed in the Amazon region (north region of Brazil, Colombia and Peru), while pacu is produced at higher latitudes in South America (southern Brazil and northern Argentina). These species have also been introduced for farming purposes in several countries in Asia, including China, Indonesia, Malaysia, Myanmar and Viet Nam^5,7^. About 142 thousand tonnes of tambaqui was produced in 2016, of which 96.4% was produced by Brazil (approximately 137 thousand tonnes)^8^. Pacu is also primarily produced in Brazil, representing the second largest native fish species production in the country, with 12 thousand tonnes produced in 2019^9^.

Despite the potential for serrasalmid species in aquaculture, their production remains entirely based on genetically unimproved stocks, and there is enormous untapped potential for selective breeding in these species. Traditional pedigree-based breeding programs have only recently been initiated for these species^10–12^. Currently, there are no reports of the incorporation of genomic information to breeding programs for serrasalmid species, which can be explained by the lack of genomic resources for this fish group. For example, only a few studies have described the discovery of novel molecular markers for these species^13–15^. However, the recent availability of genetic maps constructed using GBS (Genotyping by Sequencing) for tambaqui^16^ and RADseq (Restriction site-associated DNA sequencing) for pacu^17^, create new opportunities for the application of molecular markers to uncover the genetic basis of economically and ecologically important traits.

Genome wide association studies (GWAS) are considered useful tools for dissecting complex traits in both natural and farmed populations^18,19^. In addition, the use of genomic selection has the potential to increase genetic gain^20,21^ and, consequently, boost emerging breeding programs of new species. A few GWAS have been performed for serrasalmid species. For example, suggestive Quantitative Trait Loci (QTLs) and genes have been associated with resistance against an important bacterial pathogen in pacu^17^, and to a lack of intermuscular bones in tambaqui^22^. One of the major limitations for application of GWAS and genomic selection in serrasalmid fish is the lack of a standard high-throughput and robust genotyping assay. Dense single nucleotide polymorphism (SNP) arrays have been developed for popular farmed fish species, such as Atlantic salmon^23,24^, rainbow trout^25^, coho salmon^26^, catfish^27^, tilapia^28^ and carp^29^. These arrays have been broadly used to study traits of economic importance and to apply genomic selection in aquaculture breeding programs^30,31^.

In the present study, the main objective was to design and evaluate a multi-species SNP array for two key serrasalmid fish of economic importance, *C. macropomum* and *P. mesopotamicus*, using SNPs from multiple sources including different populations from commercial broodstocks and breeding programs. The SerraSNP array was developed using AFFYMETRIX Axiom (THERMOFISHER) technology and validated in samples from various populations of pacu and tambaqui. This dense SNP array was also tested in a closely related serrasalmid (*P. brachypomus*) to assess its potential use in other commercial species.

## Results

The sequence data used to generate the SNP dataset from ddRADseq and RADseq is part of a larger ongoing study aimed at exploring the genomic diversity of farmed tambaqui and pacu. Therefore, the descriptive data sequence of ddRADseq and RADseq will be prepared and presented in separate studies. After the de novo formation of the loci catalog and consequent read alignment, thousands of SNPs were identified in both species. Following quality-control filtering of putative SNPs, an initial selection of 130,403 and 81,848 filtered SNPs from pacu and tambaqui, respectively were identified. The in silico prediction to select the best probes according to the Axiom array criteria resulted in 99,682 and 42,851 recommended markers for pacu and tambaqui, respectively (Table 1). Further filtering step was applied to choose 30K SNPs for each species, selecting the final list of 29,612 putative SNPs for pacu and 29,575 putative SNPs for tambaqui to be included at the SerraSNP Axiom array. Most of the SNPs incorporated in the SerraSNP array were obtained by RADseq (17,932) in pacu and by ddRADseq (25,929) in tambaqui (Table 2). 2200 validated SNPs positioned in the tambaqui linkage map described by Nunes-Silva et al.^16^ passed the SNP filtering steps and were included in the final array design (Table 2).

**Table 1:** SNPs classification according the Axiom quality control criteria (*p-convert*).

Category	Number of SNPs	
	Pacu	Tambaqui
Recommended	99,682	42,851
Neutral	18,308	11,857
Not_recommended	12,413	27,140
Total	130,403	81,848

**Table 2:** SNP performance in the Axiom array, according to the method of SNP discovery in each species.

Species	Method	Putative SNPs	Selected SNPs	Polymorphic SNPs	Conversion rate	MAF
Pacu	RNA-Seq	80,004	8052	5842	0.72	0.254
	RADseq	43,355	17,932	13,345	0.74	0.175
	ddRADseq	7044	3628	2776	0.76	0.160
	Total	130,403	29,612	21,963	0.74	0.203
Tambaqui	RNA-Seq	18,282	1446	480	0.33	0.234
	GBS	6803	2200	1888	0.85	0.237
	ddRADseq	56,763	25,929	18,704	0.72	0.260
	Total	81,848	29,575	21,072	0.71	0.247

**Figure 1:**
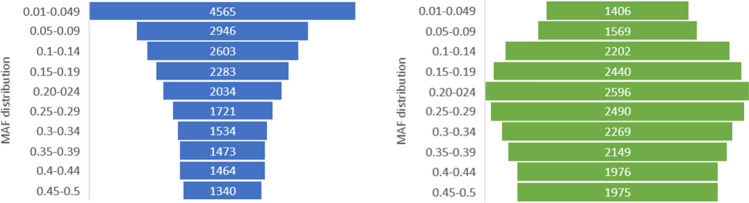
SNP distribution according to the MAF values of each species. 21,963 validated SNPs (74.17%) in pacu (blue) and 21,072 validated SNPs (71.25%) in tambaqui (green) were considered. The validation of SNPs was carried out in 94 pacu and in 58 tambaqui individuals, respectively.

The SerraSNP array was used to genotype 94 and 58 samples of pacu and tambaqui, respectively. A list containing the putative polymorphic SNPs and the flanking sequences for tambaqui and pacu were provided respectively, including: the source of sequencing and the classification based on *p-convert* values and wobble criteria (Supplementary Table S1 and Supplementary Table S2). Almost all samples passed the QC and genotype call rate > 97% threshold, except two samples of pacu and one of tambaqui. The classification of SNPs according to their quality showed that 74.17% (n = 21,963) and 71.25% (n = 21,072) were classified as polymorphic (either “Poly High Resolution” and “No Minor Hom”) in pacu and tambaqui, respectively (Table 3). The average MAF of these polymorphic SNPs in the combined total samples of pacu and tambaqui were 0.203 and 0.247, respectively (Table 4). In general, the lower MAF value in pacu is due to the larger number of SNPs distributed between MAF 0.01 and 0.049, which is mostly the result of SNPs belonging to the RADseq and ddRADseq dataset (Fig. 1). The different techniques for SNP discovery showed low interference on population segregation of SNPs (Table 4; Supplementary Table S3). Most of SNPs were segregating within each population with MAF > 0.01, ranging from 14,199 (Hatchery2) to 19,856 (Hatchery4) in pacu; and from 15,075 (Hatchery9) to 20,380 (Hatchery1) in tambaqui (Table 4).

**Table 3:** Summary of the SNP classification according to their performance in the Axiom SerraSNP array of each species.

Category	Pacu		Tambaqui	
	Number of markers	%	Number of markers	%
PolyHighResolution	16,780	56.67	19,661	66.48
NoMinorHom	5183	17.50	1411	4.77
MonoHighResolution	1771	5.98	1468	4.96
CallRateBelowThreshold	1166	3.94	1443	4.88
OffTargetVariant	570	1.92	550	1.86
Other	4142	13.99	5042	17.05
Total	29,612	100.00	29,575	100.00

**Table 4:** Descriptive population genetic estimates for the sampled pacu and tambaqui populations included in the validation of the array.

Species	Population	n	SNPs MAF > 0.01	Average MAF	H_o_	H_e_
Pacu	Hatchery 1	42	19,608	0.221	0.306	0.307
	Hatchery 2	21	18,461	0.214	0.307	0.294
	Hatchery 3	13	14,199	0.235	0.402	0.313
	Hatchery 4	18	19,856	0.210	0.285	0.292
	All populations	94	21,963	0.203	0.276	0.283
Tambaqui	Brazil—combined	28	20,380	0.266	0.362	0.356
	Hatchery 5	9	19,572	0.267	0.39	0.357
	Hatchery 6	9	17,172	0.268	0.426	0.352
	Hatchery 7	10	19,078	0.274	0.379	0.365
	Colombia—Hatchery 8	15	15,649	0.247	0.351	0.331
	Peru—Hatchery 9	15	15,075	0.248	0.353	0.329
	All populations	58	21,072	0.247	0.306	0.338
Pirapitinga	Wild	10	3042	0.278	0.454	0.348

In relation to SNPs annotation, 14,033 (66.9%) and 13,046 SNPs (62.7%) were located into transcribed regions in pacu and tambaqui, respectively (Supplementary Table S4). Regarding the RNA-Seq derived SNPs in pacu 2047 SNPs (48.3%) were in intergenic regions; 1486 (35.1%) in untranslated regions (5′ and 3′ UTR); and 705 (16.6%) in coding sequences (cds), including 490 SNPs as synonymous and 215 as missense variants (Table 5). In relation to RNA-Seq derived SNPs in tambaqui, 208 SNPs (69.8%) were identified in intergenic regions, 31 (10.4%) in 5′ and 3′ UTR; and 59 (19.8%) were considered as synonymous SNPs (Table 5).

**Table 5:** Classification of SNPs from RNA-Seq included in the array, considering the annotation of 4093 SNPs from pacu and 294 SNPs from tambaqui.

Category	Number of SNPs	
	Pacu (%)	Tambaqui (%)
Intergenic	2047 (48.3)	208 (69.8)
UTR 3′ prime	1202 (28.4)	17 (5.7)
UTR 5′ prime	284 (6.7)	14 (4.7)
**Exon**		
Synonymous SNP	490 (11.6)	59 (19.8)
Missense SNP	215 (5.0)	–
Total of effects	4238 (100)	298 (100)

Percentage values are between parenthesis.

To assess the utility of the SerraSNP array on a closely related species, which is also relevant for aquaculture, a set of DNA samples of pirapitinga (*Piaractus brachypomus*) was also tested. The samples passed the QC call rate when using a 94% threshold in the 30K SNP dataset of pacu, which demonstrates a high SNP conversion rate across this species. However, the polymorphic rate was much lower compared to pacu, resulting in 3042 SNPs (about 10% for all 30K SNPs of pacu on the SerraSNP array) (Table 4). The transferability of tambaqui markers into pacu or pirapitinga DNA resulted in failed SNP calling, even using a lower QC call rate (< 70%). The same occurred when investigating the pacu markers using tambaqui DNA samples.

Parameters of population genetics were calculated between different hatcheries of both species. The expected heterozygosity ranged between 0.29 to 0.31 in pacu and between 0.33 to 0.37 in tambaqui. The expected heterozygosity for the pirapitinga samples was 0.35. Our results indicated that populations of both species revealed high levels of genetic diversity using the parameters of MAF and heterozygosity (Table 4). Overall, no strong evidence of heterozygous deficiency was detected, and most populations had higher levels of observed heterozygosity than expected (according to HWE). In both species, the populations with highest MAF also revealed the highest heterozygosity values (Hatchery3 for pacu, and Hatchery1 for tambaqui); and, consequently, these populations have the highest genetic variability values in the present study (Table 4).

**Figure 2:**
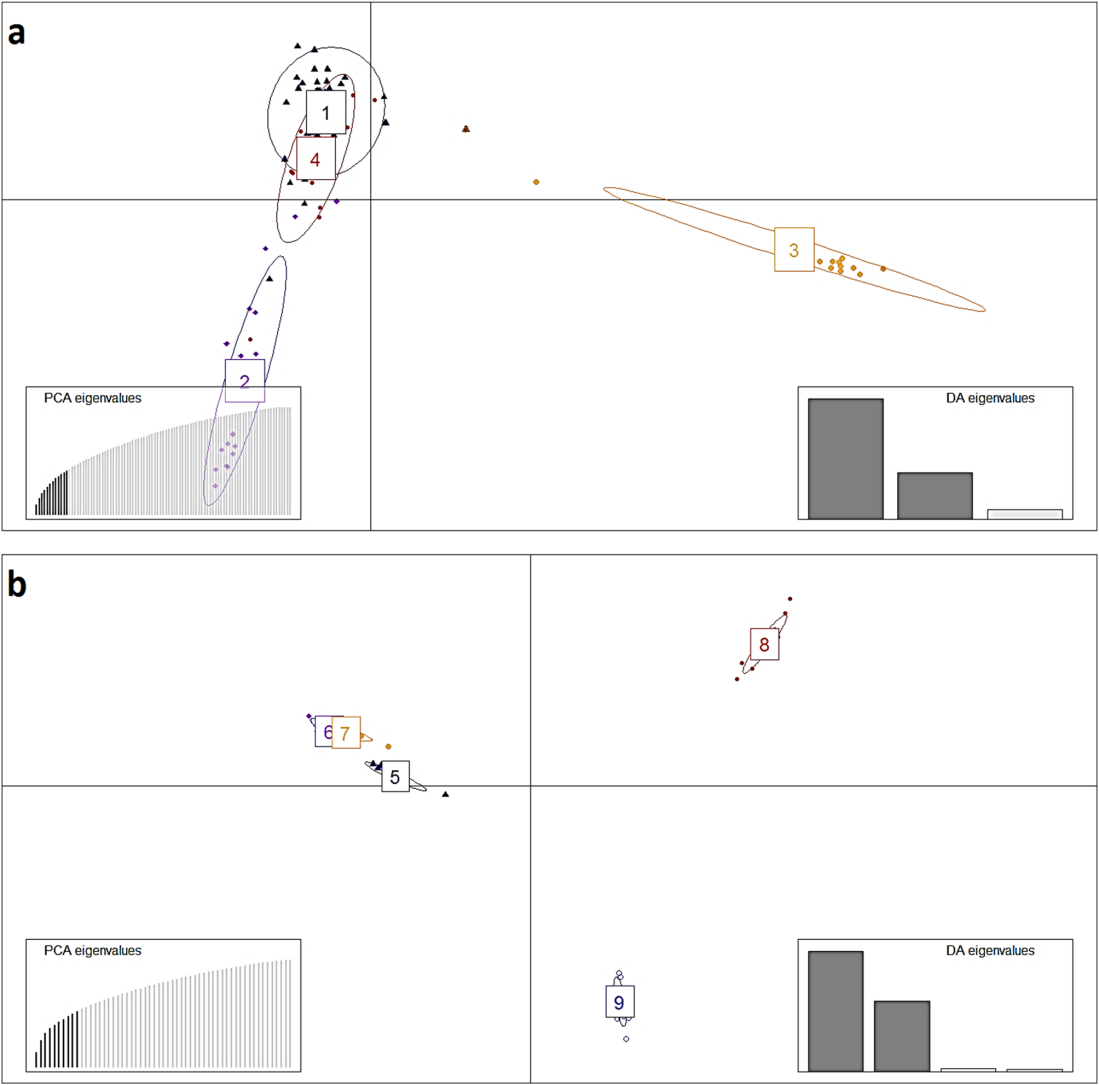
DAPC analysis from hatcheries of pacu (Hatchery 1–4) (**a**) and tambaqui (Hatchery 5–9) (**b**) to demonstrate the genetic structure using the SNPs. DAPC was performed adopting an optimum number of principal components (PC = 10) calculated using the α-score function of the Adegenet software.

According to IBS analysis for pacu (Supplementary Fig. S1) and tambaqui (Supplementary Fig. S2), and DAPC results (Fig. 2), there is evidence of the genetic structure of pacu and tambaqui samples clustering according to their hatchery origin. In pacu, hatchery2 and hatchery3 tended to cluster separately from the group formed by hatchery1 and hatchery4, which shows high genetic similarity (Supplementary Fig. S1). In tambaqui, hatchery8 (Colombia) and hatchery9 (Peru) show clear evidence of genetic differentiation in relation to populations from Brazil, which demonstrated low genetic structure to each other, particularly between hatchery6 and hatchery7 (Supplementary Fig. S2).

**Figure 3:**
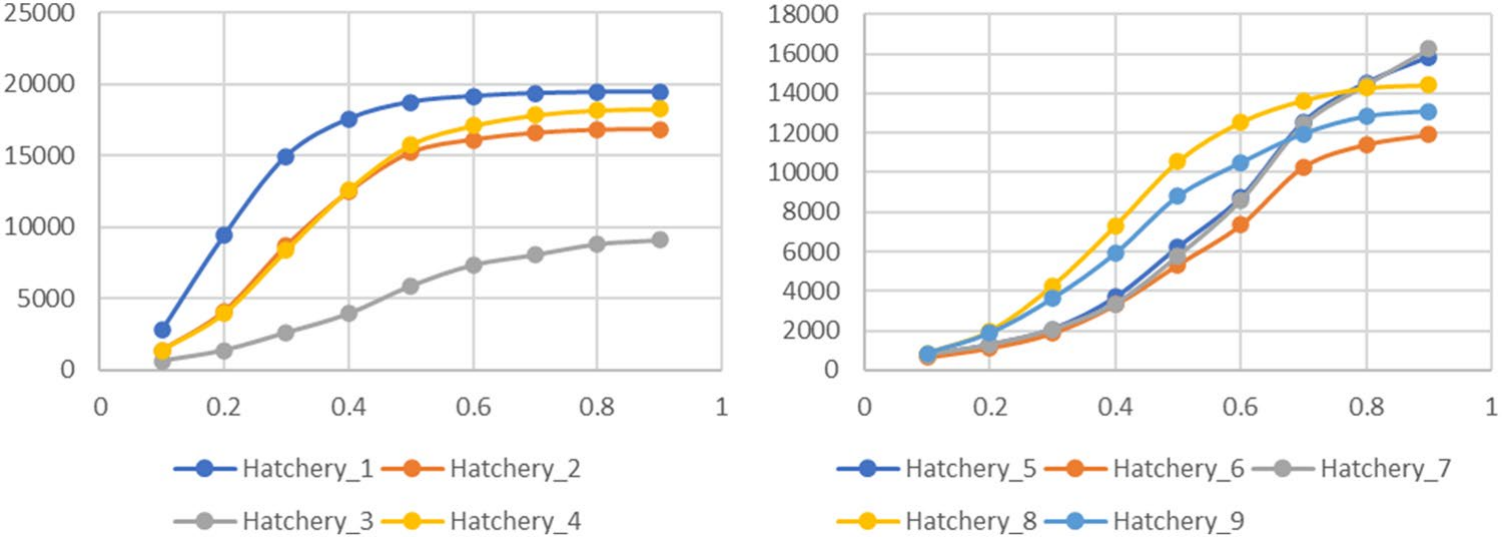
Linkage disequilibrium (LD) by SNP pruning between markers among all the sampled pacu and tambaqui populations (Hatchery 1–9). 21,963 validated SNPs (74.17%) in pacu and 21,072 validated SNPs (71.25%) in tambaqui were considered.

Due to the absence of a reference genome at chromosome level (at least not published yet) for pacu and tambaqui, the genome coverage of the polymorphic SNPs cannot be evaluated in the present study. However, correlation analysis between previously mapped SNPs included on the SerraSNP array and the corresponding linkage map for tambaqui (SNPs from GBS technique^16^) and pacu (RADseq^17^) revealed high and positive values for the relative amount (0.90 and 0.99, respectively) and density (0.98 and 0.99, respectively) (Supplementary Table S5), suggesting a wide coverage of these markers in the genome. Moreover, the extent of Linkage Disequilibrium (LD) between markers was assessed by SNP pruning for the different populations of pacu and tambaqui (Fig. 3). Pairwise r^2^ was calculated among the polymorphic SNPs (MAF > 0.01) and a range of r^2^ pruning thresholds were applied (from 0.1 to 0.9) to determine the number of markers remaining after each filtering step in each population of both species. Thus, we have a picture of the number of SNPs with different levels of redundant genotypic information. Generally, different profiles of increasing numbers of markers, at increasing levels of LD pruning, can be detected in populations of pacu. For instance, the lowest and the highest number of pruned SNPs from pacu hatchery1 and hatchery3 were at different levels of LD. Pacu hatchery2 and hatchery4 showed similar numbers of SNPs pruned at different LD levels. Assuming a r^2^ > 0.8, which is considered a strong LD threshold, the number of informative SNPs showing non-highly redundant genotypic information ranges from about 9K (hatchery3) to 19K (hatchery1), depending on the population of pacu analysed. In contrast, the number of pruned SNPs at different levels of LD from tambaqui populations, showed a similar trend between the different populations analysed. Assuming a r^2^ > 0.8, the number of informative SNPs showing non-highly redundant genotypic information ranges from about 11K (hatchery6) to 15K (hatchery5), depending on the population of tambaqui analysed.

## Discussion

Dense SNP arrays have been shown to facilitate genome-scale studies by allowing the simultaneous evaluation of thousands of SNPs in commercially important fish species, such as Atlantic salmon^23,24^, rainbow trout^25^ and tilapia^28^. These markers have facilitated the analysis of GWAS for important commercial traits (growth rate, sex determination and disease resistance) and also the implementation of genomic selection in fish species^32–35^. Although pacu and tambaqui represent the main native fish of continental aquaculture in South America, studies aimed at the incorporation of genome-wide SNP information to boost the breeding programs of these species are scarce. Therefore, the broad utility (multi-species) and open access format of the array presented here will allow the advancement of genomic studies in this fish group and support ongoing and emerging breeding programs in South America.

After assessment of the cluster properties of each of the SNPs in the SerraSNP array, most of the markers on the Axiom platform were designated as high quality and polymorphic, with validation rates of 74.17% and 71.25% in pacu and tambaqui, respectively. This conversion rate is similar to previous SNP arrays developed for other aquaculture species^36,37^, demonstrating the efficacy of our multi-strategy design and stringent filtering steps for robust SNP discovery. A balanced conversion rate was observed across the four main SNP discovery techniques, except the RNA-Seq-derived SNPs with lower conversion rate (33%) in tambaqui, which could be linked to the low coverage sequencing (454 Roche technology) and the small sampling used to generate the RNA-Seq database (*i.e.*, the previously published dataset was not sequenced with primarily target of SNP discovery), or even to the absence of a reference genome resulting in false positive markers^23^. Otherwise, the previous published GBS-derived SNPs used in tambaqui registered the highest conversion rate (85%), as result of a particular strategy in SNP discovery and validation by using linkage mapping experiments^16^. In pacu, the larger dataset of candidate SNPs provided to AFFYMETRIX were derived from RNA-Seq, but only 10% were incorporated on the Axiom array. This high discrepancy compared to RADseq and ddRADseq (40–50%) is due to the larger size of the RNA contigs, which resulted in multiple SNPs per contig; therefore, as only one SNP per contig was selected, high drop out of SNPs derived from the RNA-Seq dataset was observed.

Overall, although a high conversion rate of QC-filtered SNPs was reported, it is likely that a low proportion of false positive SNPs discovered in these sequencing experiments would remain, particularly in the ddRAD and RADseq dataset. This is due to unique potential sources of error and bias in the library preparation protocol, especially related to PCR duplicates (clonal DNA fragments originated during PCR steps) that can lead to downstream genotyping errors (for review, see Andrews et al.^38^). Moreover, false positive or failed SNPs may also occur in RNA-seq-derived SNPs because Mendelian errors in pedigreed samples were not possible to be detected by this method and/or due to limitations in the genotyping technology (SNPs located close to exon–intron boundaries).

It is also worth noting that the rate of monomorphic SNPs was low, with values of 5.98% and 4.96% in pacu and tambaqui, respectively, especially when compared to the array developed for Atlantic Salmon using similar techniques (12–53%)^23^. The high values of monomorphic SNPs (large number of false positive SNPs) in the latter study were mainly attributed to the RNA-seq technique (53%), which is particularly susceptible to false positive marker discovery^23^. Moreover, the conversion rate in the Atlantic Salmon array (46%) was relatively low comparing to the SerraSNP array (71–74%), mostly due to the duplicated genome nature in salmonids, in which was necessary to apply special strategies to avoid false positive SNPs, such as effective removal of putative paralogous variants by RR-seq (reduced-representation sequencing) of a haploid fish (conversion rate of 74%).

In the present study, a high fraction of polymorphic SNPs was identified in putative transcribed regions of the genomes of pacu and tambaqui (66.9 and 62.7%, respectively) (Supplementary Table S4). Moreover, particularly for the RNA-Seq-derived SNPs, several markers were also identified in coding sequences or regulatory regions of transcripts; therefore, they are likely to be functional and could be linked to phenotypes of economic interest. Thus, the enrichment of associated SNPs to transcribed genes in the SerraSNP array will be useful for the determining the genetic architecture of target traits in aquaculture and, consequently, for the inclusion of genomic selection in breeding programs of these species.

Population genetic analysis showed that the SNP discovery strategy used here allowed us to identify and develop a high-quality 60K genotyping array which can be reliably used to genotype different populations of farmed pacu and tambaqui. Low to moderate genetic diversity values and genetic differentiation between farmed populations have already been found for both species probably due to the lack of controlled management of broodstocks, although genetic similarity was found in between some farms most likely due to physical proximity favoring an exchange of fish between them^39,40^.

Results from the assessment of the SNP segregation between different populations indicate that the SNP panel developed in the present study would be useful for genetic applications across different populations of pacu and tambaqui, including high-resolution population genomics, genome wide-association studies and genomic selection. Nevertheless, the performance of this SNP array is expected to have a slight decrease in some populations particularly due to genetic composition of the target populations used for SNP discovery (instead ascertainment bias of the techniques). For instance, most of the samples used for SNP discovery in tambaqui were from Brazil, creating bias for Colombia and Peru hatcheries. Otherwise, a similar rate of SNPs segregation at population level was detected across the different techniques of SNP discovery, even when using limited sampling source (only 3 families by ddRAD in pacu).

The conversion rate for using SNP markers between species (pacu into tambaqui, and *vice-versa*) or using an additional species (pirapitinga) is low. This situation is comparable to the 250K SNP array for catfish, in which the transferability for different species also resulted in a low number of polymorphic SNPs^27^. Only 10% of the 30K SNPs for pacu on the SerraSNP array were polymorphic to pirapitinga; thus, the probes designed from pacu sequences could hybridize to the genomic DNA of pirapitinga, but the level of polymorphic SNPs in the latter species was limited. However, as there is no SNP marker data available for pirapitinga to date, the 3K polymorphic SNPs identified are a valuable resource for the application of genomics to population genetic and evolutionary studies, and for selective breeding in in this species; especially given the high values of genetic diversity identified (MAF = 0.28; H_o_ = 0.45 and H_e_ = 0.35).

Although a larger sample size for the population of each species for LD analysis and haplotypes construction is still necessary, the preliminary results of LD pruning demonstrate that the SNP panel of pacu and tambaqui presented here will be useful for the design of low-density SNP panels providing little redundant genotypic information. As observed previously in other fish species^41^, lower density SNP panels can be designed and applied for genomic selection and breeding, with fewer tag markers selected on interesting traits. In addition, correlation values between the mapped SNPs included in the SerraSNP array and linkage maps, previously published for tambaqui^16^ and pacu^17^, showed a high coverage and representation of the SerraSNP array throughout the genome, which reflects to its reliability and usefulness for genetic studies aiming the development of breeding programs for both species.

This study describes the development and analysis of a dense SNP array for two *Serrasalmidae* species. A large database of SNP markers using multiple approaches was developed for pacu and tambaqui, both native species widely farmed in South America. Following stringent filtering criteria, SNP assays for these two fish species were combined on the 60K SNP array, to generate a 30K high quality SNP panel for each species. Testing of the array in diverse populations revealed a high number of informative SNPs that are shared between species populations. Also, the array can be used to assess genetic diversity and population structure between populations. The SerraSNP array has open access, which will facilitate the study of important economic and ecological/evolutionary traits for these two *Serrasalmidae* species, including applications such as genomic selection, QTL mapping, phylogenetic analyses and conservation genetic programs.

## Methods

### Ethics statement

This study was conducted in strict accordance with the recommendations of the National Council for Control of Animal Experimentation (CONCEA) (Brazilian Ministry for Science, Technology and Innovation) and was approved by the Ethics Committee on Animal Use (CEUA numbers 19.005/17 and 19.006/17) of Faculdade de Ciências Agrárias e Veterinárias, UNESP, Campus Jaboticabal, SP, Brazil.

### Sample information

The tambaqui samples used for SNP discovery were obtained from broodstocks of different commercial hatcheries in South America and from the breeding nucleus of the Aquaculture Center (CAUNESP) of São Paulo State University (UNESP), Brazil (Table 6).

**Table 6:** Details about the sampling and methods used for SNP discovery for each species.

Species	Source	Method	Samples	Origin
Tambaqui	Hatchery 1—broodstock	ddRADseq^a^	11	South Brazil
	Hatchery 2—broodstock	ddRADseq^a^	17	South Brazil
	Hatchery 3—broodstock	ddRADseq^a^	17	South Brazil
	Hatchery 4—broodstock	ddRADseq^a^	15	North Brazil
	Hatchery 5—broodstock	ddRADseq^a^	18	North Brazil
	Hatchery 6—broodstock	ddRADseq^a^	21	North Brazil
	Hatchery 7—broodstock	ddRADseq^a^	15	Colombia
	Hatchery 8—broodstock	ddRADseq^a^	16	Colombia
	Hatchery 9—broodstock	ddRADseq^a^	31	Peru
	Hatchery 10—broodstock	ddRADseq^a^	8	Peru
	Breeding nucleus (Caunesp)—broodstock	ddRADseq^a^	29	South Brazil
	Breeding nucleus (Caunesp)—20 full-sib families	ddRADseq^b^	600	South Brazil
	Nunes et al.^16^—1 full-sib family	GBS	124	North Brazil
	Ariede et al.^14^—pool of 10 random individuals	RNA-Seq	10	South Brazil
	Gomes et al.^47^—pool of 8 random individuals	RNA-Seq	8	North Brazil
Pacu	Mastrochirico-Filho et al.^17^—14 full-sib families	RADseq	400	South Brazil
	Breeding nucleus (Caunesp)—3 full-sib families	ddRADseq^a^	100	South Brazil
	Mastrochirico-Filho et al.^46^—3 full-sib families	RNA-Seq	36	South Brazil

^a^Enzyme combination I (SphI and MluCI).

^b^3 families used the enzyme combination I (SphI and MluCI) and 17 families used the combination II (NlaIII and MluCI).

The commercial hatcheries represented ten different populations, of which six were from Brazil, two from Peru and two from Colombia. The samples from the Caunesp breeding nucleus were composed of 29 selected breeders, plus the parents and offspring from 20 full-sib families generated with a hierarchical mating scheme using 6 dams and 17 sires. In total, the 20 full-sib family dataset consisted of 23 parents and 577 F1 individuals (≅ 30 fish per family). The SNP array was validated with 5 different populations of tambaqui (three from Brazil, one from Colombia and one from Peru). The three from Brazil were the same populations used for SNP discovery (Table 4).

The individuals of pacu used for SNP discovery were obtained from the breeding nucleus of CAUNESP and represented 17 full-sib families (Table 6), which were generated with a hierarchical mating scheme using 8 dams and 15 sires. In total, 23 parents and 477 F1 individuals were selected from the 17 families (≅ 25 fish per family). The SNP array was tested on four different populations of pacu collected from broodstocks of different commercial hatcheries in Brazil, which were not the same populations used for SNP discovery (Table 4).

### SNP discovery and filtering

DNA extraction was performed using the DNeasy Blood & Tissue kit (QIAGEN). Purified DNA was quantified using the Qubit dsDNA BR Assay kit (INVITROGEN). There is no reference genome available for tambaqui and pacu, therefore, the ddRADseq approaches were used for SNP discovery because they are appropriate for genome reduction and de novo assembly^42,43^. Previous published databases using different techniques, such as GBS, RADseq and RNA-Seq (Table 6), were also used to increase the power of SNP discovery throughout the genome.

ddRADseq library construction was completed as previously described by Peterson et al.^43^. Briefly, 25 ng of genomic DNA from each individual was double-digested (8 U/reaction) using one of two enzyme combinations, I (NlaIII and MluCI) or II (SphI and MluCI) (Table 6), and ligated to specific adapters for each enzyme (P1 and P2, 0.25 μM) using T4 DNA ligase, at 23 °C for 1 h 30 min and 65 °C for 10 min. P1 adapters had an additional 5 nucleotides that served as individual markers (barcode). Size selection of the digested fragments was performed with E-Gel SizeSelect II (THERMOFISHER) equipment. Subsequently, PCR assays were performed to incorporate the indexes identification of each library (about 48 samples/library). Phusion enzyme conditions were used to perform the PCR assay. Reactions were purified with the AMPure XP Beads Kit and analysed by Agilent Bioanalyzer and Qubit. ddRADseq libraries were sequenced by NOVOGENE (Sacramento, USA) on ILLUMINA HiSeq4000 (PE 150 bp).

After sequencing, data were analyzed using the software package Stacks for de novo SNP identification (STACKS v. 2.41^44^). The subprograms of Stacks were implemented sequentially (*process_radtags*, *ustacks*, *cstacks*, *sstacks*). The stacks *populations* module was used to generate genotype output data for the population samples. Initially, reads were demultiplexed and filtered using *process_radtags*, with parameters that removed reads with uncalled bases, discarded reads with low quality scores and excluded sequences in which barcodes and RAD cutsites were not found. Subsequently, the loci were constructed by de novo methodology due to the absence of reference genomes for both species, using a minimum read depth of three (m = 3). A loci catalog was constructed in *cstacks* using a subset of individuals for those derived from broodstocks or using the parental individuals for those resulting from family structure. The maximum number of mismatches between sample loci when building the catalog was set to three (n = 3). The *sstacks* program was used in order to match individual putative loci (constructed by *ustacks*) against the catalog, followed by the module *populations* for SNP discovery, with the filter parameters of minor allele frequency (MAF) > 0.05 and genotyping call rate > 0.7. To differentiate putative SNPs from sequencing errors, PLINK 1.9 software^45^ was used to exclude SNPs using Hardy–Weinberg equilibrium (assessed on each individual broodstock) and Mendelian error rate (*me* 0.1 for family structure). Moreover, individuals with high missing genotype rates (*mind* 0.3) were also discarded.

The raw sequences from previous RNA-Seq databases were downloaded from the NCBI database (see Data Availability section) and the following steps were performed. The RNA-Seq database of pacu was originated from 36 individuals challenged against the bacteria *Aeromonas hydrophila* (for details, see Mastrochirico-Filho et al.^46^). The RNA-Seq raw data of tambaqui was obtained from 10 liver samples and 8 muscle and skin samples, all of them collected from random individuals, as described in detail by Ariede et al.^14^ and Gomes et al.^47^, respectively. High quality reads were used to assemble a de novo transcriptome using TRINITY v.2.9.1, adopting standard parameters^48^, such as a kmer value of 25 and contigs longer than 200 bp. The read mapping was performed by BOWTIE2 v.2.3.4.3^49^. Redundancy was reduced using the CD-HIT-EST v.4.6.8 software, clustering sequences with 95% identity^50^. After mapping, the SAMTOOLS program^51^ was used to manipulate the BAM files for SNP discovery. The filtering was performed with a quality equal to or higher than 20 (Phred score) and variants with a minimum sequencing depth of 10 reads. Abundant and repetitive SNPs in small areas were excluded. The VCFtools program^52^ was used to eliminate SNPs with MAF values less than 0.05.

SNPs from GBS were the same used for linkage mapping in tambaqui described in detail by Nunes et al.^16^.

### SNP selection for Axiom array design

A list consisting of 130,403 and 81,848 putative SNP markers for pacu and tambaqui was provided to THERMOFISHER (AFFYMETRIX) as 71-mer nucleotide sequences, with both alleles at the target SNP highlighted at position 36. A *p-convert value* (probability of a putative SNP locus converting to a reliable assay on the Axiom array) was produced by THERMOFISHER team for each submitted SNP adjacent sequence. Probes were analysed for each SNP side (forward and reverse), and then classified as recommended, neutral, or not recommended, based on p-convert values and wobble criteria (i.e., nearby interfering polymorphisms or another SNP at the flanking sequence of the target marker).

The initial analysis performed by THERMOFISHER resulted in a superior number of recommended markers than the total capacity of the Axiom MyDesign custom array. Therefore, a second step of filtering was carried out to obtain the best 30K SNPs for each species. Basically, the data was filtered to achieve only one SNP per RAD/ddRAD locus or RNA contig. When one RAD/ddRAD locus or RNA contig was identified with multiple recommended SNPs, the SNP marker with highest *p-convert* was selected.

### SNP array validation

In total, 152 genomic DNA samples (94 pacu and 58 tambaqui individuals) were sent to THERMOFISHER (USA, California) for genotyping using the SerraSNP array. The results were used to validate the performance of the array and to quantify the number of segregating SNPs in the sampled populations.

Raw data consisting of intensity calculations (CEL files) was imported into the Axiom Analysis Suite (v2.0.035, AFFYMETRIX) for genotype calling and quality control. Samples presenting a QC call rate > 0.97 and quality control (DQC) > 0.82 passed the quality control assessment (following the “Best Practices Workflow” recommended by AFFYMETRIX). Quality control analysis then classified the SNPs into categories according to their clustering performance in relation to Axiom quality control criteria: (i) “polymorphic high resolution” where the SNP passes all QC, (ii) “monomorphic high resolution” where the SNP passes all QC, but only one genotype is detected, (iii) “call rate below threshold” where genotype call rate is < 97%, (iv) “no minor homozygote” where the SNP passes all QC but only two clusters are observed, (v) “off-target variant” where atypical cluster properties arise from variants in the SNP flanking region, and (vi) “other” where the SNP does not fall into any of the previous categories. Only SNPs from categories (i) and (iv) were included and identified as polymorphic for further analyses, as they are most probably reliable and informative SNPs.

Descriptive statistics of minor allele frequencies (MAF), expected (H_e_) and observed (H_o_) heterozygosity; and IBS (Identity by State) analysis followed by multi-dimensional scaling (MDS), discriminant analysis of principal components (DAPC) and linkage disequilibrium (LD) were performed using PLINK 1.9 software^45^. A predefined tambaqui genome (not published) was adopted as reference to identify SNPs into transcribed regions using BEDOPS v2.4.40^53^. To evaluate SNPs from RNA-Seq with potential functional effects, the list of polymorphic SNPs selected for the array was annotated with SNPEff v 5.0^54^ using Uniprot proteins database as reference.

At the moment, there are still no reference genomes at chromosome level available for both species. However, linkage maps were previously developed for tambaqui^16^ and pacu^17^ using 7192 and 17,453 SNPs, respectively. Of these total, 25.6% and 42.6% of mapped SNPs were included respectively in the SerraSNP array, and were correlated to the linkage maps to evaluate the level of coverage and genetic representativeness of the SerraSNP array for both species.

## Supplementary Information

Supplementary Figure S1.

Supplementary Figure S2.

Supplementary Table S1.

Supplementary Table S2.

Supplementary Table S3.

Supplementary Table S4.

Supplementary Table S5.

## Data Availability

The raw fastq files obtained by RADseq methods for SNP discovery in tambaqui^55^ and pacu^56^ are available in the National Center for Biotechnology Information (NCBI) Sequence Read Archive (SRA). RNA sequencing information is encompassed for pacu^57^ and tambaqui^58^ by NCBI BioProject ID PRJNA632934 and PRJNA358254, respectively.

## Code availability

Parameters of software involved in the analysis are described in the following paragraph. FastQC: v0.11.8, standard parameters. RADseq methods: (1) Stacks: v. 2.0, m = 3, n = 3, r = 0.70. (2) Plink: v. 1.9, -hwe 10^–6^ –me 0.1 0.9 -mind 0.3. RNA-Seq method: (1) Trimmomatic: v.0.36, LEADING:20 TRAILING:20 SLIDINGWINDOW:4:20 MINLEN:36. (2) Trinity: v.2.9.1, standard parameters kmer-length value of 25 and contiguous sequences (contigs) longer than 200 bp. (3) BWA (Burrows-Wheeler Aligner): v. 0.7.17, mem algorithm. (4) SAMtools programs: version 1.9, default parameters, vcfutils.pl varFilter -d 10 -a 3.
