# Supplementary figures and images for: Development of a multi-species SNP array for serrasalmid fish Colossoma macropomum and Piaractus mesopotamicus
Source: Sci Rep. 2021 Sep 29;11:19289. doi: 10.1038/s41598-021-98885-x (PMC8481427; doi:10.1038/s41598-021-98885-x)

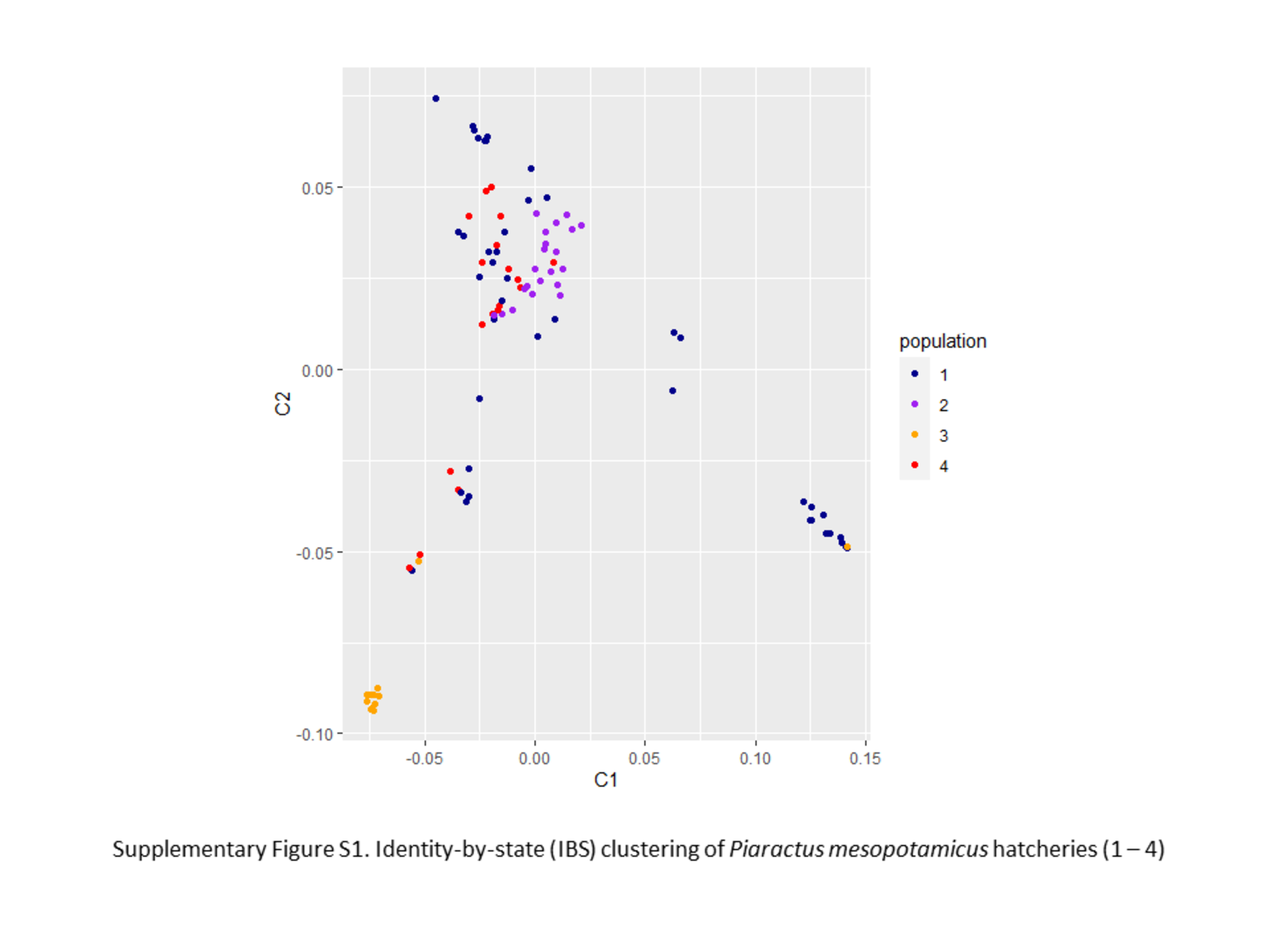

Supplement: Supplementary file 1 — Supplementary Figure S1. [file 41598_2021_98885_MOESM1_ESM.tiff]

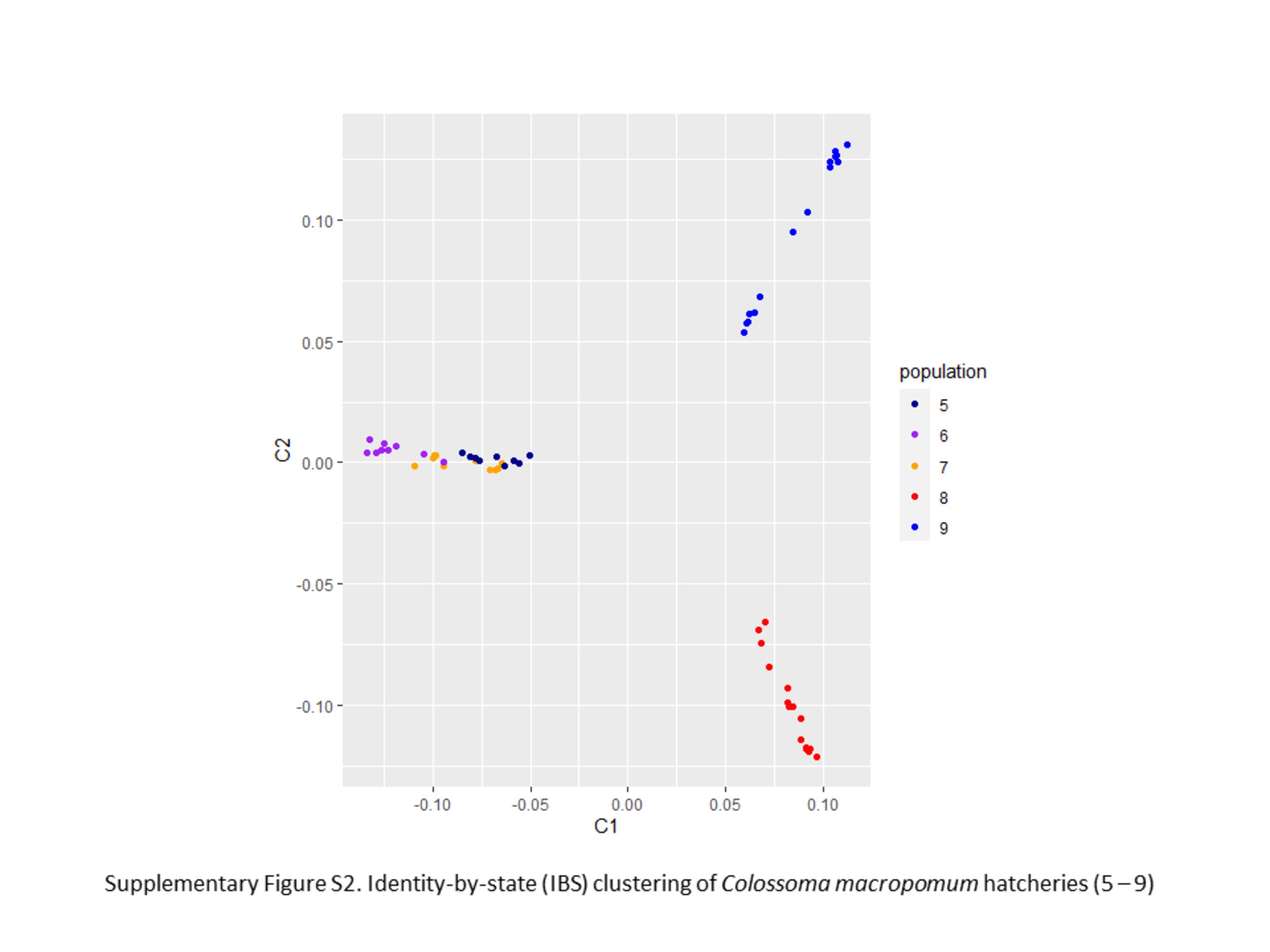

Supplement: Supplementary file 2 — Supplementary Figure S2. [file 41598_2021_98885_MOESM2_ESM.tiff]
